# Supplementary material for: Identification of photoperiod-induced specific miRNAs in the adrenal glands of Sunite sheep (Ovis aries)
Source: Front Vet Sci. 2022 Jul 22;9:888207. doi: 10.3389/fvets.2022.888207 (PMC9354845; doi:10.3389/fvets.2022.888207)
Supplement: Supplementary file 1 [file Data_Sheet_1.ZIP › Supplementary file/miRDeep2 file.zip!/miRDeep2 file/Novel_Structure/SPLP42A2/Novel_16.pdf]

Provisional ID	:	NC_019484.2_43617
Score total	:	1.7
Score for star read(s)	:	-1.3
Score for read counts	:	0
Score for mfe	:	1.4
Score for randfold	:	1.6
Score for cons. seed	:	
Total read count	:	21
Mature read count	:	20
Loop read count	:	0
Star read count	:	1

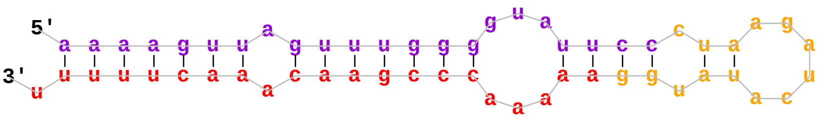

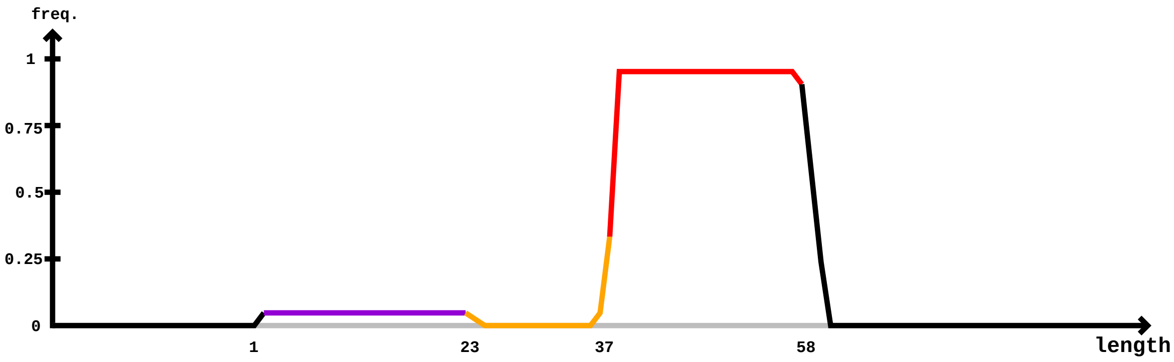

Star Mature

```
5'- aauuuuauacuggguuggccaaaaguuaguuugggguauucccuaagaucauaugg aaaaacccgaacaaacuuuuuggcaaacccaauaguuucuuacaggacucaacgaa   -3'   obs
    aauuuuauacuggguuggccaaaaguuaguuugggguauucccuaagaucauauggaaaaacccgaacaaacuuuuuggcaaacccaauaguuucuuacaggacucaacgaa         exp
    ..........((((((.((((((((((.(((((((...((((.((......)).))))...))))))).))).))))))).))))))..(((((......)))))....... reads   mm   sample
    ....................aaaaguuaguuugggguauucc......................................................................  1       0    seq
    .......................................................gaaaaaccUgaacaaacuuuu....................................  1       1    seq
    ........................................................aaaaacccgaacaaacuuuuu...................................  3       0    seq
    ........................................................aaaaacccgaacaaacuuuuG...................................  1       1    seq
    ........................................................aaaaacccgaacaaacuuuuugA.................................  2       1    seq
    .........................................................aaaaUccgaacaaacuuuuu...................................  3       1    seq
    .........................................................aaaaccUgaacaaacuuuuu...................................  2       1    seq
    .........................................................aaaaUccgaacaaacuuuuug..................................  3       1    seq
    .........................................................aaaaccUgaacaaacuuuuug..................................  2       1    seq
    .........................................................aaaaccUgaacaaacuuuuugg.................................  2       1    seq
    .........................................................aaaaUccgaacaaacuuuuugg.................................  1       1    seq
```
